# Heat for wounds – water-filtered infrared-A (wIRA) for wound healing – a review

**DOI:** 10.3205/000235

**Published:** 2016-06-29

**Authors:** Gerd Hoffmann, Mark Hartel, James B. Mercer

**Affiliations:** 1 Institute of Sports Sciences, Johann Wolfgang Goethe University Frankfurt/Main, Frankfurt/Main, Germany; 2 Department of Surgery, Klinikzentrum Mitte, Dortmund, Germany; 3 Cardiovascular Research Group, Department of Medical Biology, Institute of Health Sciences, Faculty of Medicine, University of Tromsø, Tromsø, Norway

**Keywords:** water-filtered infrared-A (wIRA), wound healing, acute and chronic wounds, reduction of pain, tissue oxygen partial pressure, tissue temperature

## Abstract

**Background:** Water-filtered infrared-A (wIRA) is a special form of heat radiation with high tissue penetration and a low thermal load to the skin surface. wIRA corresponds to the major part of the sun’s heat radiation, which reaches the surface of the Earth in moderate climatic zones filtered by water and water vapour of the atmosphere. wIRA promotes healing of acute and chronic wounds both by thermal and thermic as well as by non-thermal and non-thermic cellular effects.

**Methods:** This publication includes a literature review with search in PubMed/Medline for “water-filtered infrared-A” and “wound”/”ulcus” or “wassergefiltertes Infrarot A” and “Wunde”/”Ulkus”, respectively (publications in English and German), and additional analysis of study data. Seven prospective clinical studies (of these six randomized controlled trials (RCT), the largest study with n=400 patients) were identified and included. All randomized controlled clinical trials compare a combination of high standard care plus wIRA treatment vs. high standard care alone. The results below marked with “vs.” present these comparisons.

**Results: **

- wIRA increases tissue temperature (+2.7°C at a tissue depth of 2 cm), tissue oxygen partial pressure (+32% at a tissue depth of 2 cm) and tissue perfusion (effect sizes within the wIRA group).
- wIRA promotes normal as well as disturbed wound healing by diminishing inflammation and exudation, by promotion of infection defense and regeneration, and by alleviation of pain (with respect to alleviation of pain, without any exception during 230 irradiations, 13.4 vs. 0.0 on a visual analogue scale (VAS 0–100), median difference between groups 13.8, 95% confidence interval (CI) 12.3/16.7, p<0.000001) with a substantially reduced need for analgesics (52–69% less in the three groups with wIRA compared to the three control groups in visceral surgery, p=0.000020 and 0.00037 and 0.0045, respectively; total of 6 vs. 14.5 analgesic tablets on 6 surveyed days (of weeks 1–6) in chronic venous stasis ulcers, median difference –8, 95% CI –10/–5, p=0.000002).

Further effects are:

- Faster reduction of wound area (in severely burned children: 90% reduction of wound size after 9 vs. 13 days, after 9 days 89.2% vs. 49.5% reduction in wound area, median difference 39.5% wound area reduction, 95% CI 36.7%/42.2%, p=0.000011; complete wound closure of chronic venous stasis ulcers after 14 vs. 42 days, median difference –21 days, 95% CI –28/–10, p=0.000005).
- Better overall evaluation of wound healing (surgical wounds: 88.6 vs. 78.5 on a VAS 0–100, median difference 8.9, 95% CI 6.1/12.0, p<0.000001).
- Better overall evaluation of the effect of irradiation (79.0 vs. 46.8 on a VAS 0–100 with 50 as neutral point, median difference 27.9, 95% CI 19.8/34.6, p<0.000001).
- Higher tissue oxygen partial pressure during irradiation with wIRA (at a tissue depth of 2 cm 41.6 vs. 30.2 mmHg, median difference 11.9 mmHg, 95% CI 9.6/14.2 mmHg, p<0.000001).
- Higher tissue temperature during irradiation with wIRA (at a tissue depth of 2 cm 38.9 vs. 36.4°C, median difference 2.6°C, 95% CI 2.2/2.9°C, p<0.000001).
- Better cosmetic result (84.5 vs. 76.5 on a VAS 0–100, median difference 7.9, 95% CI 3.7/12.0, p=0.00027).
- Lower wound infection rate (single preoperative irradiation: 5.1% vs. 12.1% wound infections in total, difference –7.0%, 95% CI –12.8%/–1.3%, p=0.017, of these: late wound infections (postoperative days 9-30) 1.7% vs. 7.7%, difference –6.0%, 95% CI –10.3%/–1.7%, p=0.007).
- Shorter hospital stay (9 vs. 11 postoperative days, median difference –2 days, 95% CI –3/0 days, p=0.022).

Most of the effects have been proven with an evidence level of 1a or 1b.

**Conclusion:** Water-filtered infrared-A is a useful complement for the treatment of acute and chronic wounds.

## Zusammenfassung

**Hintergrund:** Wassergefiltertes Infrarot A (wIRA) ist eine spezielle Form der Wärmestrahlung mit hohem Eindringvermögen in das Gewebe und geringer thermischer Belastung der Hautoberfläche. wIRA entspricht dem Großteil der die Erdoberfläche in gemäßigten Klimazonen durch Wasser und Wasserdampf der Atmosphäre gefiltert erreichenden Sonnenwärmestrahlung. wIRA fördert die Heilung akuter und chronischer Wunden sowohl über thermische und temperaturabhängige als auch über nicht-thermische und temperaturunabhängige zelluläre Effekte.

**Methoden:** Diese Publikation schließt eine Literaturübersicht mit Suche in PubMed/Medline nach “water-filtered infrared-A” und “wound”/”ulcus” oder “wassergefiltertes Infrarot A” und “Wunde”/”Ulkus” (Publikationen in Englisch und Deutsch) und zusätzliche Analysen von Studiendaten ein. 7 prospektive klinische Studien (davon 6 randomisierte kontrollierte Studien (RCT), die größte Studie mit n=400 Patienten) wurden gefunden und eingeschlossen. Alle randomisierten kontrollierten klinischen Studien vergleichen eine Kombination aus Therapie auf hohem Niveau plus wIRA-Therapie vs. Therapie auf hohem Niveau allein. Die mit „vs.“ gekennzeichneten Ergebnisse unten zeigen diese Vergleiche.

- wIRA steigert die Temperatur (+2,7°C in 2 cm Gewebetiefe) und den Sauerstoffpartialdruck im Gewebe (+32% in 2 cm Gewebetiefe) und die Gewebedurchblutung (Größe der Effekte innerhalb der wIRA-Gruppe).
- wIRA fördert sowohl die normale als auch die gestörte Wundheilung, indem es Entzündung und Sekretion mindert, Infektionsabwehr und Regeneration fördert und Schmerzen lindert (bezüglich Schmerzlinderung ausnahmslos während 230 Bestrahlungen, 13.4 vs. 0,0 auf einer visuellen Analogskala (VAS 0–100), mediane Differenz zwischen den Gruppen 13.8, 95%-Konfidenzinterval (KI) 12.3/16.7, p<0,000001) mit relevant weniger Analgetikabedarf (52–69% weniger in den drei Gruppen mit wIRA verglichen mit den drei Kontrollgruppen in der Viszeralchirurgie, p=0,000020 bzw. 0,00037 bzw. 0,0045; insgesamt 6 vs. 14,5 Schmerztabletten an 6 Erhebungstagen (der Wochen 1–6) bei chronischen venösen Ulzera, mediane Differenz –8, 95%-KI –10/–5, p=0,000002).

Weitere Effekte sind:

- Schnellere Wundflächenabnahme (bei schwerbrandverletzten Kindern 90% Wundflächenabnahme nach 9 vs. 13 Tagen, nach 9 Tagen 89,2% vs. 49,5% Wundflächenabnahme, mediane Differenz 39,5% Wundflächenabnahme, 95%-KI 36,7%/42,2%, p=0,000011; kompletter Wundschluss bei chronischen venösen Ulzera nach 14 vs. 42 Tagen, mediane Differenz –21 Tage, 95%-KI –28/–10, p=0,000005).
- Bessere Gesamteinschätzung der Wundheilung (Operationswunden: 88,6 vs. 78,5 auf einer VAS 0–100, mediane Differenz 8,9, 95%-KI 6,1/12,0, p<0,000001).
- Bessere Gesamteinschätzung des Effekts der Bestrahlung (79,0 vs. 46,8 auf einer VAS 0–100 mit 50 als Neutralpunkt, mediane Differenz 27,9, 95%-KI 19,8/34,6, p<0,000001).
- Höherer Gewebe-Sauerstoffpartialdruck während Bestrahlung mit wIRA (in 2 cm Gewebetiefe 41,6 vs. 30,2 mmHg, mediane Differenz 11,9 mmHg, 95%-KI 9,6/14,2 mmHg, p<0,000001).
- Höhere Gewebetemperatur während Bestrahlung mit wIRA (in 2 cm Gewebetiefe 38,9 vs. 36,4°C, mediane Differenz 2,6°C, 95%-KI 2,2/2,9°C, p<0,000001).
- Besseres kosmetisches Ergebnis (84,5 vs. 76,5 auf einer VAS 0–100, mediane Differenz 7,9, 95%-KI 3,7/12,0, p=0,00027).
- Niedrigere Wundinfektionsrate (einzelne präoperative Bestrahlung: 5,1% vs. 12,1% Wundinfektionen insgesamt, Differenz –7,0%, 95%-KI –12,8%/–1,3%, p=0,017, von diesen: späte Wundinfektionen (an den postoperativen Tagen 9 bis 30) 1,7% vs. 7,7%, Differenz –6,0%, 95%-KI –10,3%/–1,7%, p=0,007).
- Kürzerer Krankenhausaufenthalt (9 vs. 11 postoperative Tage, mediane Differenz –2 Tage, 95%-KI –3/0 Tage, p=0,022).

Die meisten Effekte sind mit einem Evidenzgrad von 1a bzw. 1b belegt.

**Schlussfolgerung:** wIRA ist eine nützliche Ergänzung für die Behandlung von akuten und chronischen Wunden.

## Introduction

Wound healing is often a clinical challenge, as wound pain, non-healing wounds with a variety of pathogenetic factors, and a limited wound healing velocity, even in undisturbed wound healing, decrease quality of life and cause tremendous costs, especially in chronic wounds [1]. A variety of strategies, methods or substances including wound dressings are used to overcome such problems. Beside negative pressure wound therapy and hyperbaric oxygenation the application of heat belongs to the group of technical methods for improving wound healing. Positive effects with pre-operative [2] and post-operative [3] warming of the operative field have been shown. 

Water-filtered infrared-A (wIRA), as a special form of heat radiation (in the range 780–1400 nm) with a high tissue penetration and a low thermal load to the surface of the skin, is a substance-free non-contact, pleasant feeling method, which can be used in both acute and chronic wounds.

## Methodological aspects

This review presents an overview of seven prospective clinical studies, of which six were randomised controlled (RCTs), and other experiences related to the application of wIRA for the improvement of healing in acute and chronic wounds (reviews: [4], [5], [6], [7], [8], [9], [10], [11], [12], [13], [14]). 

The main aim is to compare a combination of high standard care plus wIRA treatment vs. high standard care alone in both acute and chronic wounds in humans. Several different main variables of interest were observed, including amount of pain, necessary pain medication, evaluation of wound healing, cosmetic aspect, velocity of wound healing, reduction of wound area, wound infection rate, tissue oxygen partial pressure, tissue temperature, and final overall evaluation of the effect of irradiation (including pain, wound healing, and cosmetic result). Central element of this review is Table 1 (Tab. 1), presenting 13 main variables of interest. 

**Figure 1:**
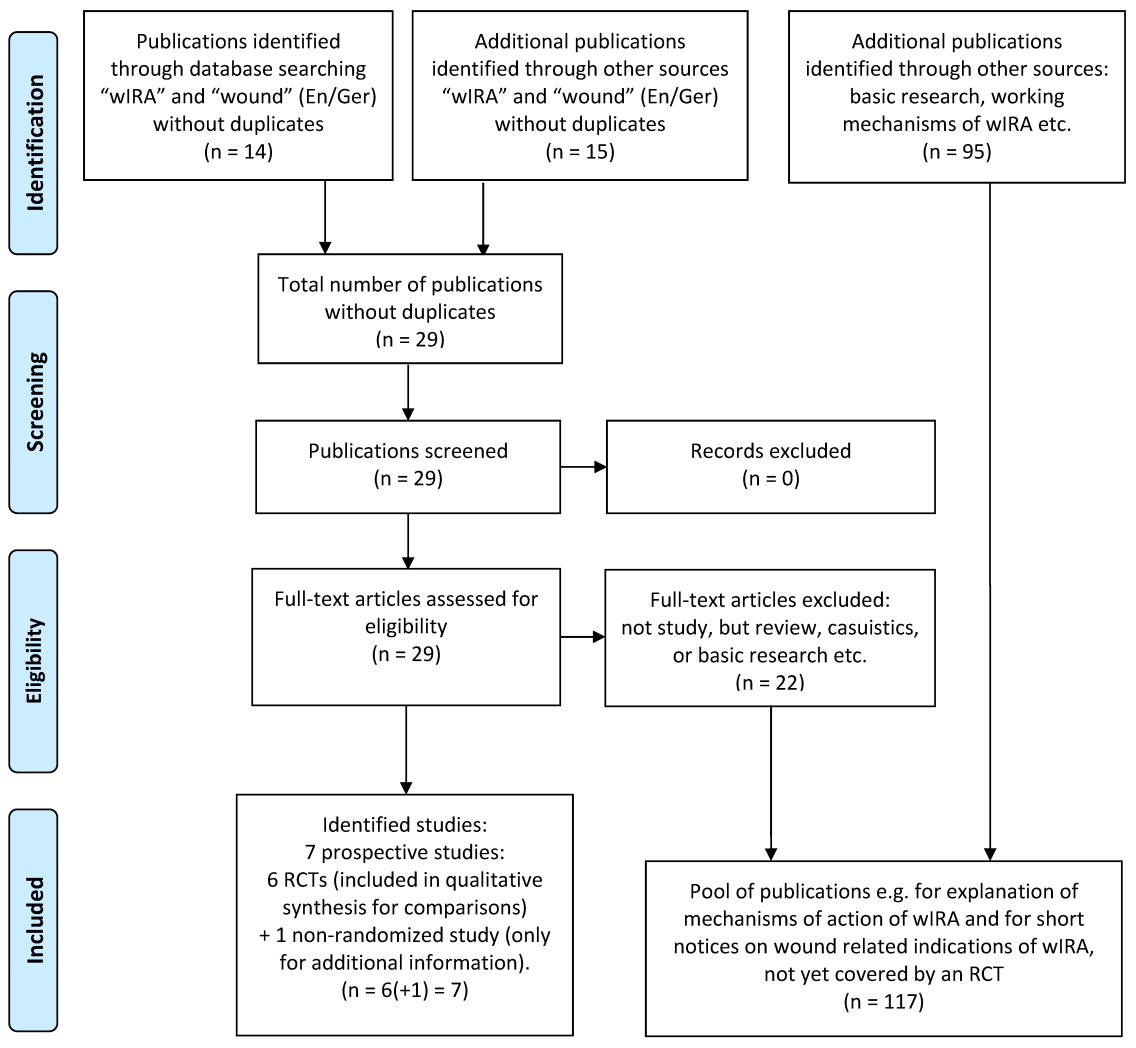
Flow diagram for literature search and study selection (adapted from [16])

Publications for this review were identified by searches in PubMed/Medline for “water-filtered infrared-A” and “wound”/”ulcus” or “wassergefiltertes Infrarot A” and “Wunde”/”Ulkus”, respectively (publications in English and German up to August 2015) and manual searches in reference lists, see flow diagram, Figure 1 (Fig. 1). All clinical studies met the inclusion criteria and were included (with restrictions of use concerning one non-randomized study). All clinically relevant variables of interest were extracted from the full text of the publications. Clinically relevant basic research has also been taken into account, e.g. for explanations concerning mechanisms of action of wIRA. Wound related indications for wIRA, not yet covered by an RCT, are briefly mentioned using appropriate literature. 

Concerning additional clinical fields for the application of wIRA besides wound healing we refer to the three broader reviews [12], [13], [14].

Non-parametric and parametric statistics should not be mixed. Non-parametric statistics do not have any prerequisites concerning distribution (they are independent from distribution or “distribution-free”) and are always permissible to be used. With regard to the skewed distributions, which are often seen in medical data (e.g. duration of hospital stay), non-parametric (distribution-free) methods and characteristic variables were used [15]. In accordance with the PRISMA statement concerning the preparation of reviews [16] – besides median values of the groups – as “effect estimates and confidence intervals” median differences between the groups and confidence intervals were used. In case median differences between the groups and confidence intervals were not presented in the original publication, they were calculated from the original data provided by the authors of the respective studies (studies Heidelberg, Kassel, and Basel) or derived from data obtained from the publication (study Munich) using “BiAS for Windows, version 11.0, Hochheim, Darmstadt, Germany” [17]. The Hodges-Lehmann estimator for the difference between groups (median of differences between groups, “median differences” in Table 1 (Tab. 1)) was used, which offers additional information compared to the sole presentation of the medians of the groups and the difference of the medians. For confidence intervals the 95% or 99% Moses confidence interval for the Hodges-Lehmann estimator for the difference between groups was used. Due to different study designs and different observed variables meta-analysis could not be performed. However, an evidence synthesis was possible just by reporting the corresponding results of the trials.

The “VAS 0–100” are 100 mm visual analogue scales on paper forms (open bars, 100 mm long, without scaling, only the endpoints are marked with “0” and “100” and a corresponding verbal description; in cases with 50 as neutral point, this point is marked as well), see an example in reference [18]. The visual analogue scales are continuous scales with infinite possible expressions. Using a ruler, the individual expression can be classified into one of 101 steps (0–100).

Some additional methodological details are presented in the legend of Table 1 (Tab. 1).

In addition to the 7 reported clinical studies and other publications personal clinical experience in the application of wIRA for wound healing since 1991 was included, especially concerning limitations of the studies or concerning fields not yet covered by clinical studies. 

## Principles of water-filtered infrared-A (wIRA)

**Figure 2:**
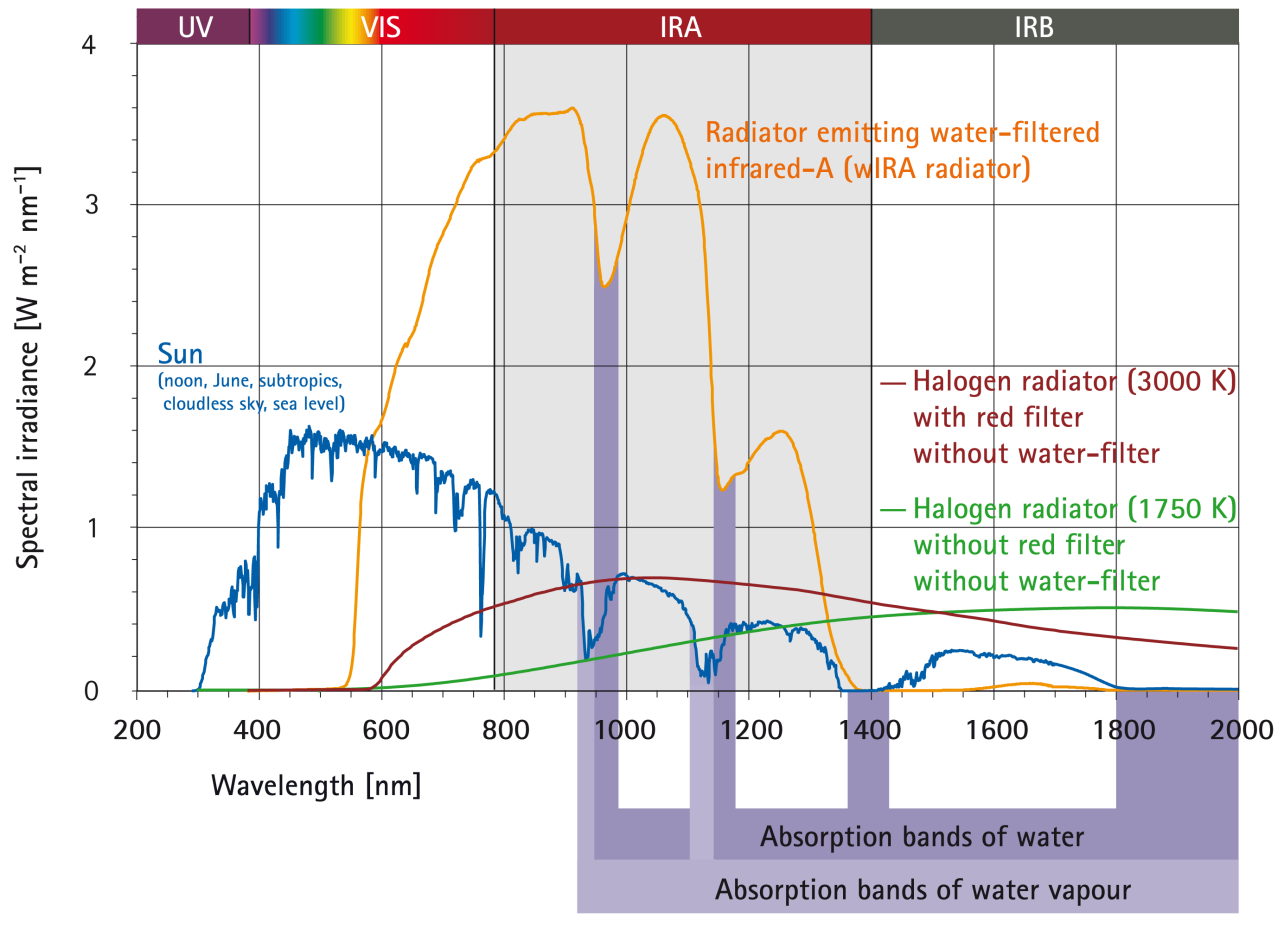
Comparison of the spectra of a radiator with water-filtered infrared-A (wIRA) and of the sun measured under a cloudless sky in June at noon at sea level in the subtropics and of two different halogen radiators without water-filter for therapeutic and wellness applications (with kind permission of Dr. Helmut Piazena, Charité Berlin; from [29]). The presented irradiances of the wIRA radiator and of the two different halogen radiators cause the same skin surface temperature rise in humans (temperature-related equivalence of the irradiations). The presented solar irradiance is near the maximum possible value in the subtropics at noon in midsummer on the surface of the Earth at sea level with cloudless sky. The relations between the four presented spectra are therefore realistic. A typical wIRA radiator emits no ultraviolet radiation (UV) and almost no infrared-B and infrared-C radiation (less than 0.5% compared to 50–80% infrared-B and infrared-C in conventional infrared radiators without water-filter) (details in [29]).

In moderate climatic zones, in contrast to desert regions, the thermal radiation from the sun is perceived at the Earth’s surface as being pleasantly warm, causing no stinging or burning sensations in the skin due to the filtering of the radiation by water and water vapour of the atmosphere [6], [12], [19]. The filter effect of water decreases those parts of infrared radiation (most parts of infrared-B and -C and the absorption bands of water within infrared-A), which would otherwise, by reacting with water molecules in the skin, cause an undesired thermal load to the surface of the skin (Figure 2 (Fig. 2)) [4], [6], [19], [20], [21], [22]. 

Technically, water-filtered infrared-A is produced by special radiators. Typically, the complete non-coherent and non-polarized broadband radiation of a 3000 Kelvin halogen bulb is passed through a cuvette containing water, which absorbs or decreases the described undesired wavelengths of the infrared radiation (Figure 2 (Fig. 2); additional figures in reference [4]) [12], [23]. The remaining wIRA radiation (in the range 780–1400 nm) has a high penetration capacity in tissue so that in comparison to conventional unfiltered infrared radiation a considerably higher amount of energy can be transferred deeply into the tissue while the thermal load to the skin surface remains low [4], [24], [25]. Thermography shows different skin surface temperature with the same total irradiance: a water-filtered infrared-A radiator causes a lower skin surface temperature than conventional infrared radiators without water-filter [4]. With equal skin surface temperature the total irradiance of infrared-A of a water-filtered infrared-A radiator is nearly 4–9-fold compared to conventional infrared radiators without water-filter. For certain clinically relevant wavelengths, such as 820 nm [26], [27], [28], the irradiance can be even greater (approximately 6–30-fold, see Figure 2 (Fig. 2)) [5], [29], [30], [31]. 

A typical wIRA radiator emits no ultraviolet radiation (UV) and almost no infrared-B and infrared-C (less than 0.5% compared to 50–80% in conventional infrared radiators without water-filter) [4], [5], [24], [25], [29], [30], [31], [32]. Approximately 73% of the total irradiance of a typical wIRA radiator are in the range infrared-A. Only 33% of the total irradiance of the sun, reaching the surface of the Earth, are in the range infrared-A, as the total irradiance of the sun is much more dominated by visible light (380–780 nm) (Figure 2 (Fig. 2)) [4], [29], [30].

In contrast to water-filtered infrared-A, an irradiation of a wound with unfiltered infrared would lead to stinging and burning and drying of the wound, thus limiting the tolerable irradiance and making its use unethical. 

Detailed information of the principles and the mechanisms of action of wIRA can be found in [6] and [12]. 

## Mechanisms of action of wIRA

The effects of wIRA are based on both its thermal effects (relying on transfer of heat energy) and thermic effects (temperature-dependent effects, occurring together with temperature changes) as well as on non-thermal and temperature-independent effects [4], [6], [12], [33], [34]. 

wIRA generates a therapeutically usable thermal field in tissue which is detectable down to a depth of approximately 5 cm [6], [35]. Under clinical conditions, acute increases of tissue temperature, oxygen partial pressure and perfusion have been measured. The following increases (within the wIRA group) were found: temperature at the surface of the skin by almost 6°C from 32.5°C to 38.2°C [36] and at a depth of 2 cm by 2.7°C [4], [37], tissue oxygen partial pressure by 10 mmHg (32%) at a tissue depth of 2 cm [4], [37], and blood perfusion in tissue down to a depth of 5 cm [9], [35]. In addition, the blood flow at the skin surface can increase to 8-fold [36]. (Concerning temperature and oxygen partial pressure, both at a tissue depth of 2 cm, Table 1 (Tab. 1) presents the comparisons between wIRA group and control group with partly higher effects of wIRA.)

Obviously, wound healing is an energy consuming process and energy production in tissue depends on an adequate supply of oxygen and energy containing substrates. Therefore, tissue temperature, oxygen partial pressure and perfusion are crucial factors for improving the supply of energy and oxygen to tissue [4], [6], [12], [18], [37], [38], [39], [40], [41], e.g. a 10°C higher temperature approximately doubles the reaction velocity and the energy production (reaction velocity temperature rule). Wound healing and resistance to infections (e.g., granulocyte function including formation of antibacterial oxygen radicals) depend on a sufficient supply of energy and oxygen [4], [6], [12], [18], [37], [38], [39], [40], [41], [42]. In particular, chronic wounds are often extremely hypoxic [4], [6], [18], [37], [38], [39], [40], [41], [43], which increases the risk of infections considerably [37], [39], [42]. An improvement of energy supply per unit of time (increase of metabolic activity) and in the oxygen supply therefore provides a plausible explanation for the positive clinical effects of wIRA in wounds and wound infections [6], [12], [18], [37], [44]. 

Additionally, wIRA and infrared-A radiation have non-thermal effects which occur without notable increases in temperature [4], [12], [45], [46], [47], [48], [49], [50], [51], [52], [53], [54], [55]. Non-thermal effects of wIRA, e.g. on cytochrome c oxidase, are described in detail in reference [12]. These non-thermal effects include a stimulation of wound healing [56] through a direct stimulation of cells and cellular structures or substances, e.g., cytochrome c oxidase [28], [57], [58], [59], [60], which enhances mitochondrial function and triggers protective functions [12], [61]. 

Irradiation with visible light (VIS) and wIRA presumably acts with endogenous protoporphyrin IX (or protoporphyrin IX of bacteria) in a manner similar to a mild photodynamic therapy (endogenous PDT-like effect) [4], [8], [44], [55]. This could lead to improved cell regeneration and wound healing and to prevention of infections and to antibacterial effects [4], [8], [44], [55].

wIRA in therapeutic irradiation intensities and doses has been shown not only to be harmless to human skin [6], [34], [53], [54], but also to have cytoprotective effects [6], [34], [49], [51], [52], [53], [54], [62]. These aspects have been extensively discussed in references [6], [34] and [24]. The application of wIRA with appropriate irradiation intensities can be considered as being safe [6], [7], [8], [24], [32], [34], [63], [64].

## Application of wIRA

**Figure 3:**
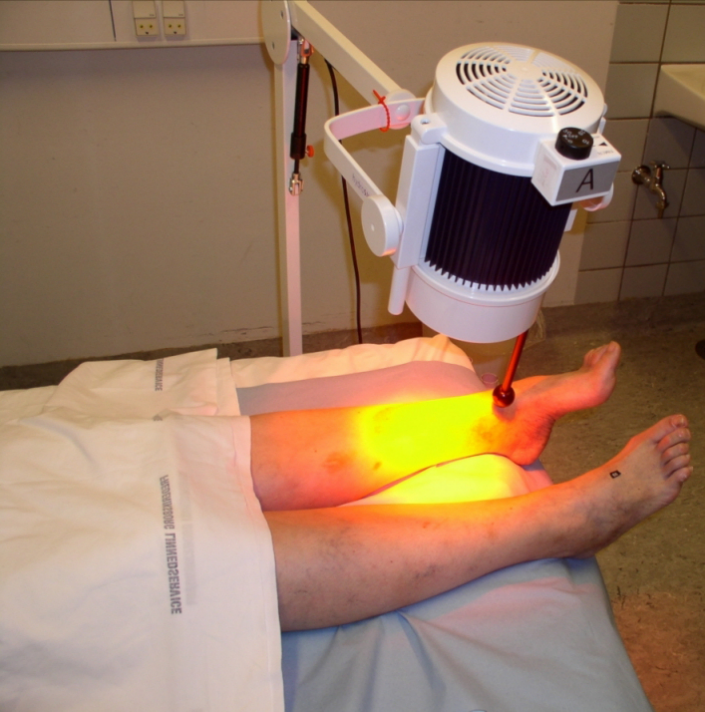
Example of an irradiation of a wound with a radiator for water-filtered infrared-A (wIRA) (from [6], [18])

The irradiation of uncovered skin or wounds is carried out with a wIRA radiator positioned perpendicularly to the skin [4] and without the use of expendable materials (see Figure 3 (Fig. 3)). This treatment is applied 1–2 times per day, with each irradiation lasting at least 20–30 min or for considerably longer periods of time (several hours). The irradiation should be administered with moderate intensity which is perceived as being pleasant (typical irradiance of 60–120 mW/cm² wIRA or 80–160 mW/cm² wIRA with visible light (VIS)). Depending on the type of radiator this corresponds to the radiator being positioned at a distance of 30–55 cm from the skin. The irradiance can be reduced by simply increasing the distance of the radiator from the skin so that the patient does not feel the irradiation to be too warm [5], [6]. Typically distances up to approximately 75 cm or even more can be used [5], [6], [34]. In patients with an impaired sensorium (e.g., patients with a diabetic polyneuropathy), an impaired ability to respond, inadequately perfused tissue, cold tissue, or thin subcutaneous tissue (e.g. along the tibia ridge), a lower irradiation intensity should be used by increasing the radiator-to-patient distance [5], [6]. Undesirable effects (at worst burns) can be avoided by always using moderate irradiancies. 

Typically wIRA effects can be increased by using longer daily irradiation times (concerning wounds e.g. 2–5 hours per day), while keeping the irradiation intensity moderate [5]. With moderate irradiances the irradiation time is only limited by practical aspects. 

Suitable in-patients can be trained in proper and safe use regarding the prescribed scheme of irradiation by a physician or other trained medical staff. Such patients can afterwards administer wIRA in the hospital by themselves with e.g. keeping the wIRA radiator near their beds. This can help to keep costs using wIRA small. 

After getting advice in proper and safe use of wIRA suitable patients can as well easily apply wIRA at home by themselves [65]. This allows long daily irradiation times and use of wIRA even at weekends and avoids the necessity of visiting a physician or a physiotherapist with a wIRA radiator for each treatment, thereby saving both time and money.

## Clinical effects of wIRA in wounds

Table 1 (Tab. 1) presents an overview of the most important clinical effects of wIRA in acute and chronic wounds with a level of evidence of 1a/1b according to the “Oxford Centre for Evidence-based Medicine – Levels of Evidence (March 2009)” [66] (OCEBM).

Clinically, wIRA is capable of substantially reducing pain, both during a single wIRA irradiation and also, in the medium term, with a considerable reduction in the need for analgesics (e.g., 52–69% lower, median differences) (Table 1 (Tab. 1)) [4], [12], [18], [37].

The pain reduction achieved by wIRA can be explained by thermal effects such as accelerated wound healing, increased elimination and metabolisation of accumulated metabolites, pronounced muscle-relaxing effects, as well as via non-thermal effects (possibly on nociceptors) [7], [9], [12], [37]. Pain reduces perfusion and oxygen partial pressure [67]. Vice versa a reduction in pain [68] increases the oxygen partial pressure [7], [37], [69] and thus reduces the risk of infections considerably [7], [9], [12], [37], [39], [42], [70], [71]. The pain reduction achieved from wIRA treatment occurs in a wide range of indications including back pain [14].

wIRA is capable of accelerating wound healing both in acute as well as in chronic wounds (including infected wounds) or can improve an impaired wound healing (Table 1 (Tab. 1)) [4], [6], [7], [8], [9], [12], [18], [37], [65]. The reduction of the wound area or complete wound closure can be accelerated (Table 1 (Tab. 1)) [4], [7], [65]. Healing has been achieved in previously recalcitrant wounds [4], [18], [65]. Not only the disturbed wound healing, but even the normal, unimpeded wound healing process can be improved by wIRA (Table 1 (Tab. 1)) [4], [7], [12], [37].

wIRA is capable of reducing wound secretion and inflammation (Table 1 (Tab. 1)) [4], [12], [18], [37], [65]. The reduction of inflammation and secretion/exudation (or an increase in the resorption of fluids in cases of fluid accumulation such as seromas [4], [7], analogue to an enhancement in the resorption of topically applied substances [72], [73]) through wIRA has been described for a wide spectrum of indications (e.g., reduction of wound exudation or bronchial secretion) [12]. 

**Figure 4:**
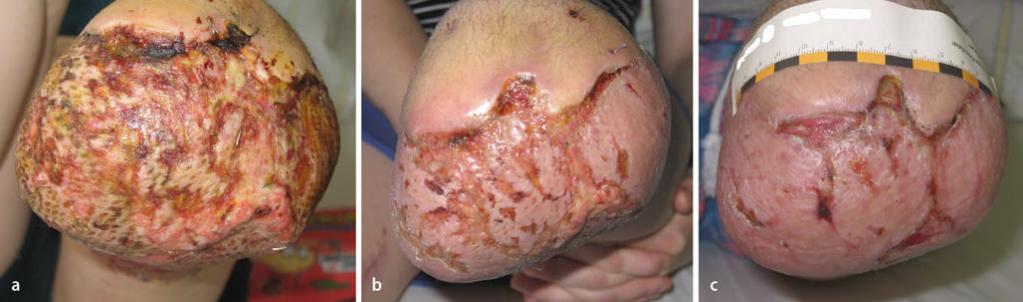
a: Progressive disintegration of transplanted split-skin graft caused by infection with “extended spectrum” β-lactamase (ESBL) forming Klebsiella species, view of the stump crest (femur), 9 days postoperatively. b: 4 days after transfer and beginning with the irradiations with wIRA, 3 times 1 hour per day. c: Progressive epithelialisation 3 weeks after beginning with the irradiations with wIRA. (from [5])

An example of a treatment course of a wound with wIRA is given in Figure 4 (Fig. 4).

## Therapy of acute wounds with wIRA

### wIRA postoperatively for acute surgical wounds 

**Figure 5:**
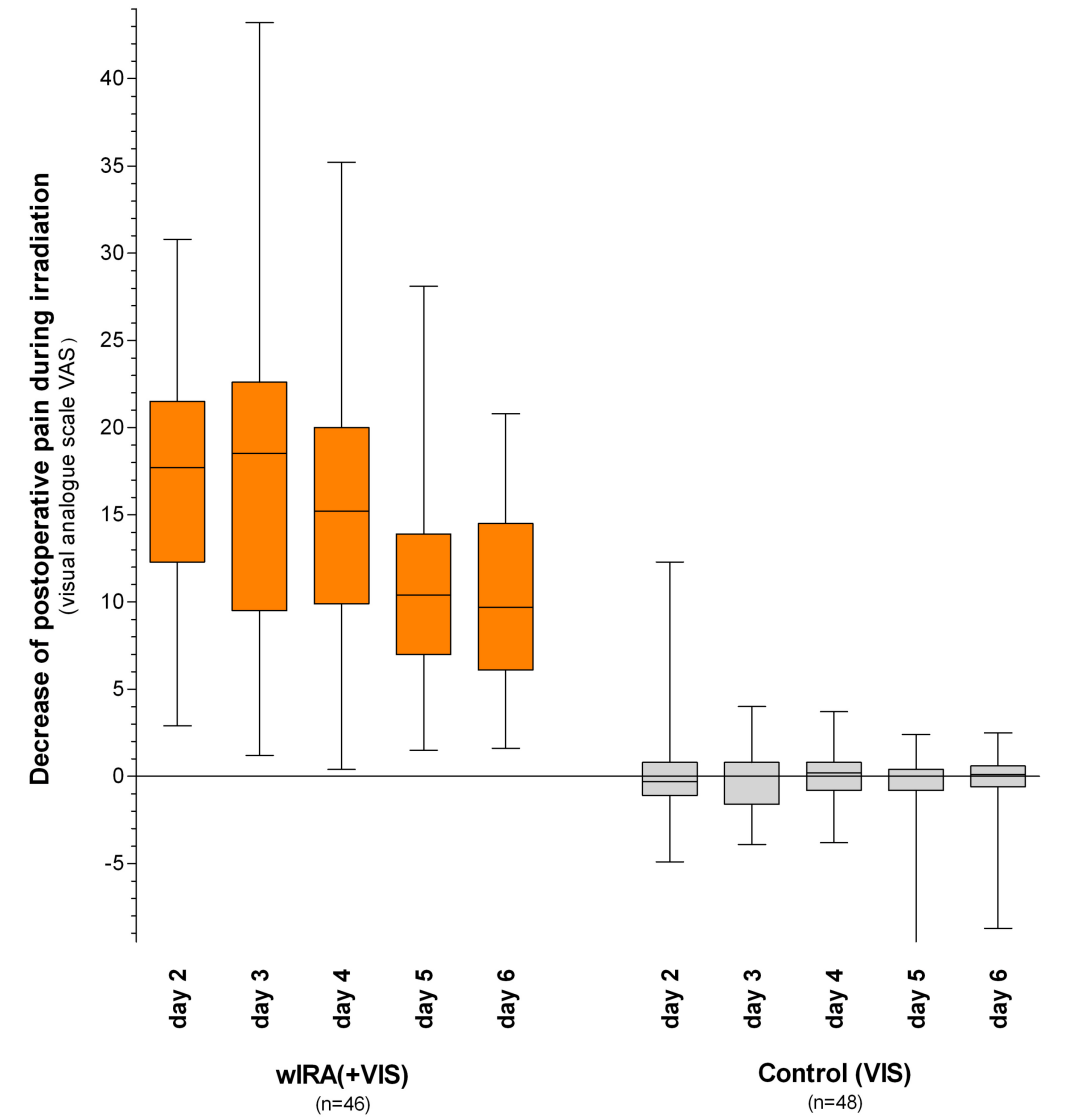
Decrease of postoperative pain during irradiation in the group with water-filtered infrared-A (wIRA) and visible light (VIS) and in the control group with only visible light (VIS) (abdominal operations, Study Heidelberg) Pain was assessed using a visual analogue scale VAS 0–100; values are presented as minimum, 25%-percentile, median, 75%-percentile and maximum (box and whiskers graph with the box representing the interquartile range; from [4], adapted from [7], [37]). In 230 single irradiations with wIRA(+VIS) there was, without any exceptions, a decrease in the pain score between the start and the end of the 20 minute period of treatment, while pain scores remained unchanged in the control group (p<0.000001 for any single documented day as well as for all of the days taken together).

**Figure 6:**
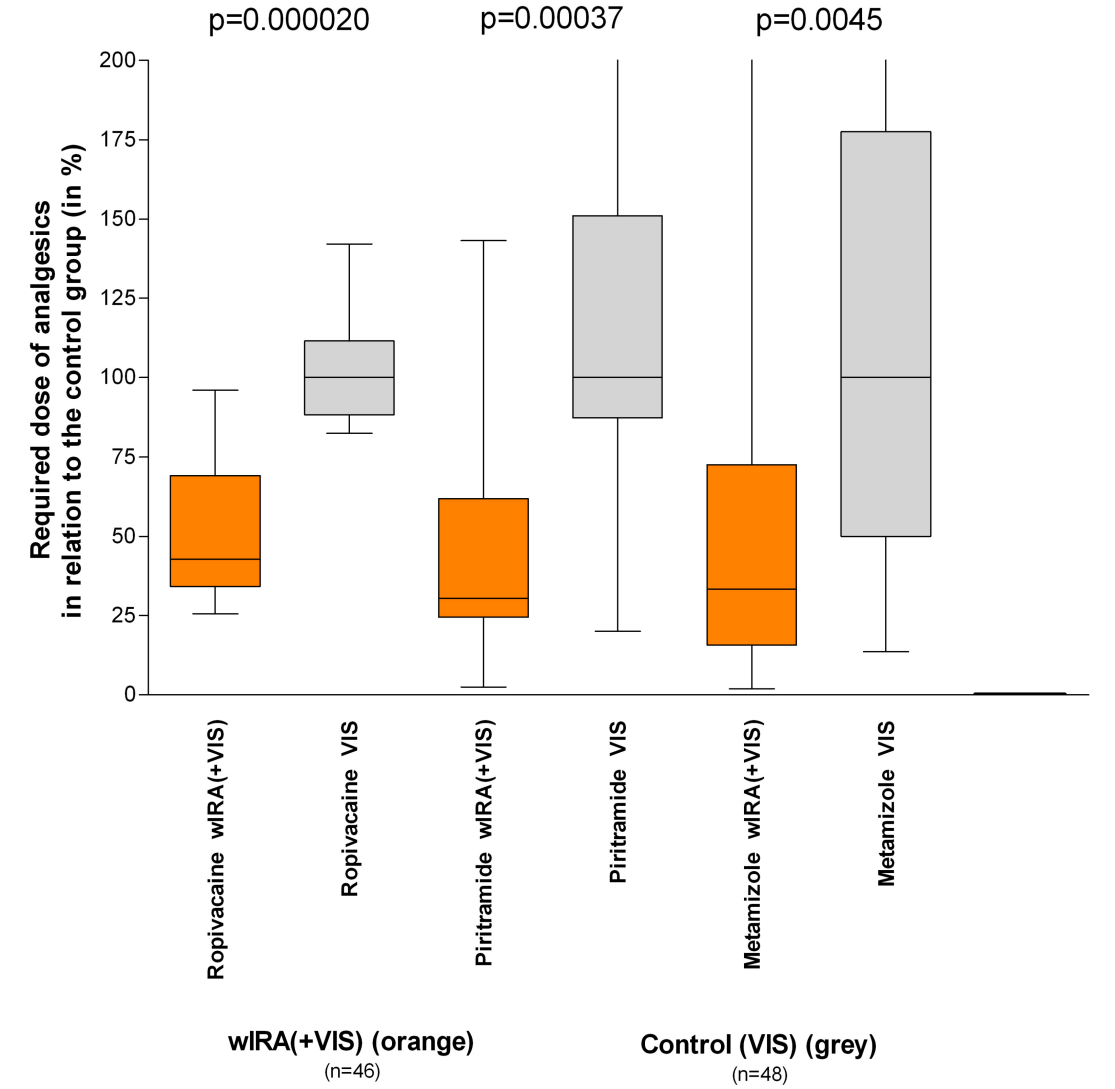
Required dose of analgesics of the subgroups with water-filtered infrared-A (wIRA) and visible light (VIS) in relation to the control subgroups with only visible light (VIS) (medians of the control subgroups = 100) (Study Heidelberg) (values are presented as minimum, percentiles of 25, median, percentiles of 75, and maximum (box and whiskers graph with the box representing the interquartile range), from [4], adapted from [7], data taken from [37]). The required dose of analgesics was 52–69% lower (median differences) in the subgroups with wIRA(+VIS) compared to the control subgroups with only VIS.

A prospective, randomised, controlled double-blind study with 111 patients who had undergone major abdominal operations (Department of Surgery, University Heidelberg, Germany) [4], [12], [37] revealed that 20 minutes of irradiation twice per day (starting on the second postoperative day) in the group with wIRA and visible light VIS (with a maximum of 175 mW/cm² wIRA und 45 mW/cm² VIS) resulted in a significant and relevant reduction of pain (without any exception in 230 irradiations) compared to the control group with only VIS (evaluation of 94 patients per protocol, Table 1 (Tab. 1) and Figure 5 (Fig. 5)). This was associated with a decrease in the required dose of analgesics (52–69% less, median differences) (Table 1 (Tab. 1) and Figure 6 (Fig. 6)). 

**Figure 7:**
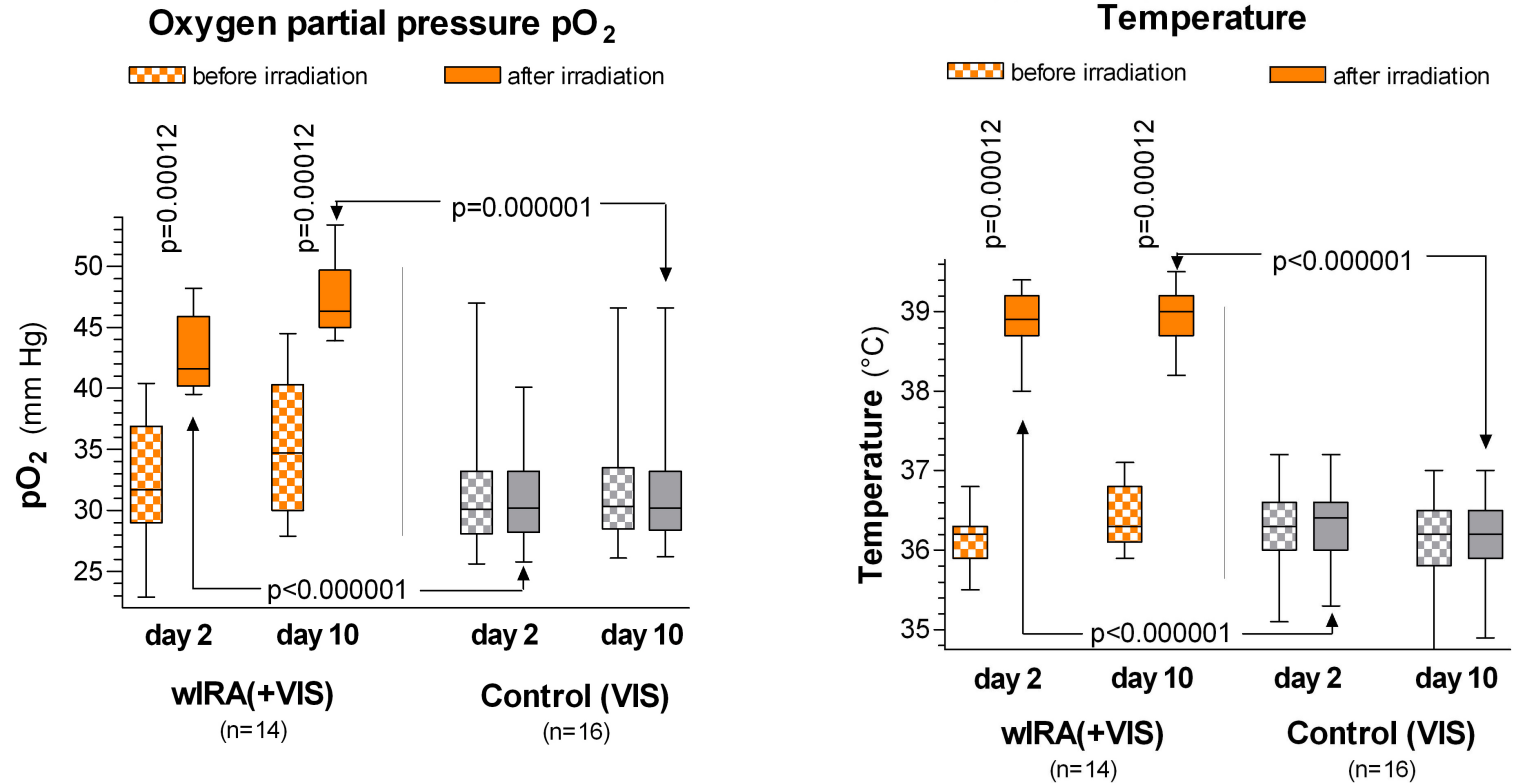
Tissue oxygen partial pressure (left) and tissue temperature (right) measured at a tissue depth of 2 cm on the postoperative days 2 and 10 in the group with water-filtered infrared-A (wIRA) and visible light (VIS) and in the control group with only visible light (VIS) (abdominal operations, Study Heidelberg). Values are presented as minimum, 25%-percentile, median, 75%-percentile and maximum (box and whiskers graph with the box representing the interquartile range; from [4], adapted from [7], [37]). During irradiation with wIRA(+VIS), the tissue oxygen partial pressure rose markedly by more than 30% and the tissue temperature rose markedly by approximately 2.7°C, whereas both variables remained unchanged in the control group.

During irradiation with wIRA(+VIS) the tissue oxygen partial pressure rose by 10.3 mmHg (+32%) and the tissue temperature by 2.7°C (both measured at a tissue depth of 2 cm), whereas both variables in the control group remained unchanged. For details of the comparison between the groups (p<0.000001, significant) see Table 1 (Tab. 1) and Figure 7 (Fig. 7). Both variables showed effects, which reached beyond the time span of the single irradiation: the pre-irradiation values increased from the second to the tenth postoperative day. 

The final overall evaluation of the effect of irradiation (including wound healing, pain and cosmetic result), which was assessed by the surgeon and the patient using a VAS (0–100, with 50 representing the indifference point where no effect was seen) showed much better results for the wIRA group than for the control group (Table 1 (Tab. 1)). A similar finding was observed for the single aspects, evaluation of wound healing (surgeon: p<0.000001, significant) and the cosmetic result (Table 1 (Tab. 1)). The evaluation of wound healing took into account the following factors: erythema, oedema, increasing local skin temperature, seroma, and haematoma. Additionally, a trend towards a lower incidence of wound infections and a shorter postoperative hospital stay was found (Table 1 (Tab. 1)). 

The main result of the study was that postoperative irradiation with wIRA was capable of improving the disturbed as well as even the normal unimpeded wound healing process [4], [37].

The results of the study are in accordance with earlier publications showing positive effects with pre-operative [2] and post-operative [3] warming of the operative field with other heating methods.

### wIRA preoperatively for acute surgical wounds 

In a prospective, randomised, controlled double-blind study (Department of Surgery, Technical University Munich, Germany) a single, 20-minute irradiation with wIRA and visible light (VIS) was carried out immediately prior to surgery in patients who underwent major abdominal operations; primarily 400 patients were included in the study [70]. The study in Munich confirmed findings of the above mentioned study in Heidelberg, where patients were irradiated *postoperatively*, especially the marked decrease of the total wound infections with *5.1%* (9 of 178) vs. *12.1%* (22 of 182) (difference –7.0%, p=0.017, evaluation of 360 patients per protocol (full-analysis set)). Of these, especially the late wound infections (postoperative days 9–30) were markedly decreased in the wIRA group: *1.7%* (3 of 178) vs. *7.7%* (14 of 182) (difference –6.0%, p=0.007). The overall endured VAS pain score in the recovery phase (until day 30 postoperatively) tended to smaller pain scores in the wIRA group compared to the control group. 

### wIRA for severely burned children 

**Figure 8:**
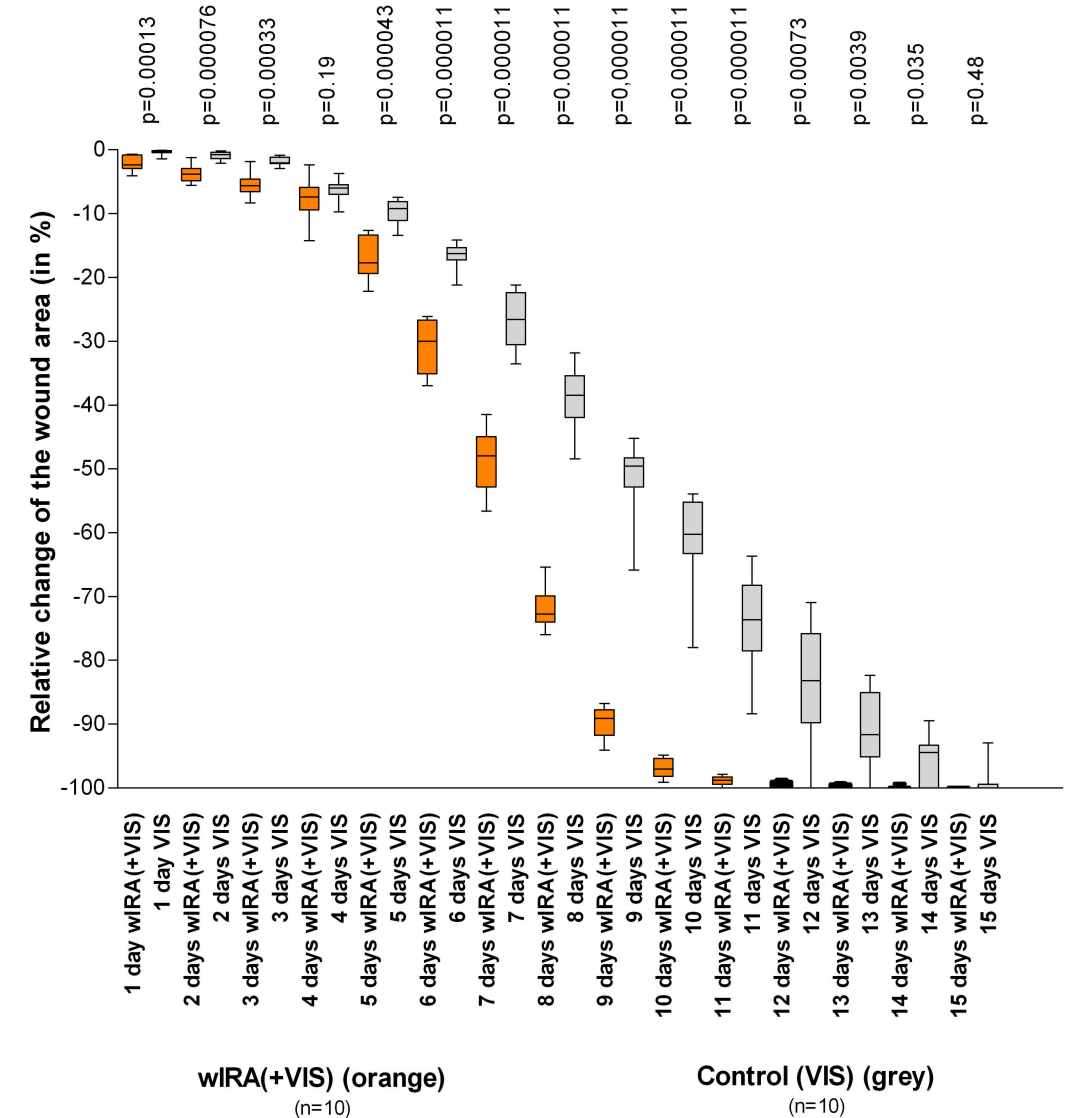
Relative change of wound area in severely burned children as a function of duration of treatment (in days) in the group with water-filtered infrared-A (wIRA) and visible light (VIS) and in the control group with only visible light (VIS) (Study Kassel) Values are presented as minimum, 25%-percentile, median, 75%-percentile and maximum (box and whiskers graph with the box representing the interquartile range; from [4], adapted from [7]). The figure presents the data from those 10+10 = 20 children (out of 21+24 = 45 children), who had second degree, type a burns (not second degree, type b burns) and who were consequently treated non-surgically until complete cutaneous regeneration occurred including irradiation (starting on the day of the burn, until complete reepithelialization) with wIRA(+VIS) or with only VIS (control group). Patients in the group with wIRA showed a markedly faster reduction of wound area compared to the control group: a median reduction of wound size of 50% was reached in the group with wIRA after 7 days compared to 9 days in the control group, a median reduction of wound size of 90% was achieved in the group with wIRA after 9 days compared to 13 days in the control group.

A prospective, randomised, controlled, double-blind study in 45 severely burned children (Department of Paediatric Surgery, Children’s Hospital Park Schoenfeld, Kassel, Germany) [4], [7], [12] showed that 30 minutes irradiation daily (starting on the day of burn) with wIRA and VIS resulted in considerably faster reduction of the wound area as compared to a control group with only VIS. On the fifth day (after 4 days with irradiation), the decision was taken as to whether surgical debridement of necrotic tissue was necessary because of deeper (second degree, type b) burns or whether non-surgical treatment was possible (second degree, type a burns; 10 of 21 in the group with wIRA, 10 of 24 in the control group). The patients treated conservatively were kept within the study and irradiated until reepithelialisation was complete. After 9 days 89.2% (in the group with wIRA+VIS) versus 49.5% (in the control group with VIS only) reduction of the wound area were achieved, p=0.000011 (significant) (Table 1 (Tab. 1) and Figure 8 (Fig. 8)). 

In addition, the group receiving wIRA showed better results in terms of the overall surgical evaluation of the wounds compared to the control group [4].

### wIRA and experimental human wounds 

In a prospective, randomised controlled study with 12 volunteers (Department of Dermatology, University Medical Center Charité Berlin, Germany) [7], [12], [74] four experimental superficial wounds (5 mm in diameter) were created in each volunteer using the suction blister technique together with removal of the roof of the blister using a scalpel and sterile forceps (day 1). Four different treatments were applied over 10 days and assessed: No therapy; wIRA(+VIS) (applied daily for 30 minutes); dexpanthenol ointment once daily; wIRA(+VIS) with dexpanthenol ointment once daily. 

The regeneration of the Stratum corneum (from the first layer of corneocytes to full development) was determined using laser scan microscopy. The fastest regeneration (in particular for days 5–7) occurred in the group using the combination wIRA(+VIS) with dexpanthenol ointment. This was followed by wIRA(+VIS) alone, then dexpanthenol ointment alone, with the slowest regeneration being found in the untreated wounds [7], [12], [74].

### wIRA for wound seromas and for persistent postoperative pain 

For these indications only observations but no randomised controlled trials are so far available: During rehabilitation after hip or knee endoprosthetic operations the resorption of wound seromas and wound haematomas was both clinically and sonographically faster and pain was reduced by irradiation with wIRA(+VIS). Therefore, wIRA presents a non-invasive alternative to punction and wound revision (for details see [7], [12]).

wIRA can also impressively reduce persistent postoperative pain, e.g., after thoracotomy [7].

## Therapy of chronic wounds with wIRA

### wIRA for chronic venous stasis ulcers of the lower legs 

In a prospective, randomised, controlled study all 40 patients received standard care for chronic venous stasis ulcers of the lower legs (initial ulcer area 1–12.4 cm²) in Basel, Switzerland [8], [10], [12]. 20 patients were additionally treated with wIRA(+VIS) 30 minutes three times per week over maximally 6 weeks. This treatment regime resulted in a relevant and significant acceleration of wound healing (14 versus 42 days until complete wound closure, p=0.000005) compared to the control group with the same standard care without irradiation (Table 1 (Tab. 1)). In addition there was a significant and relevant reduction of the required dose of analgesics (6 versus 14.5 tablets in total over the 6 days of assessment in the weeks 1–6, p=0.000002) compared to the control group (Table 1 (Tab. 1)). 

**Figure 9:**
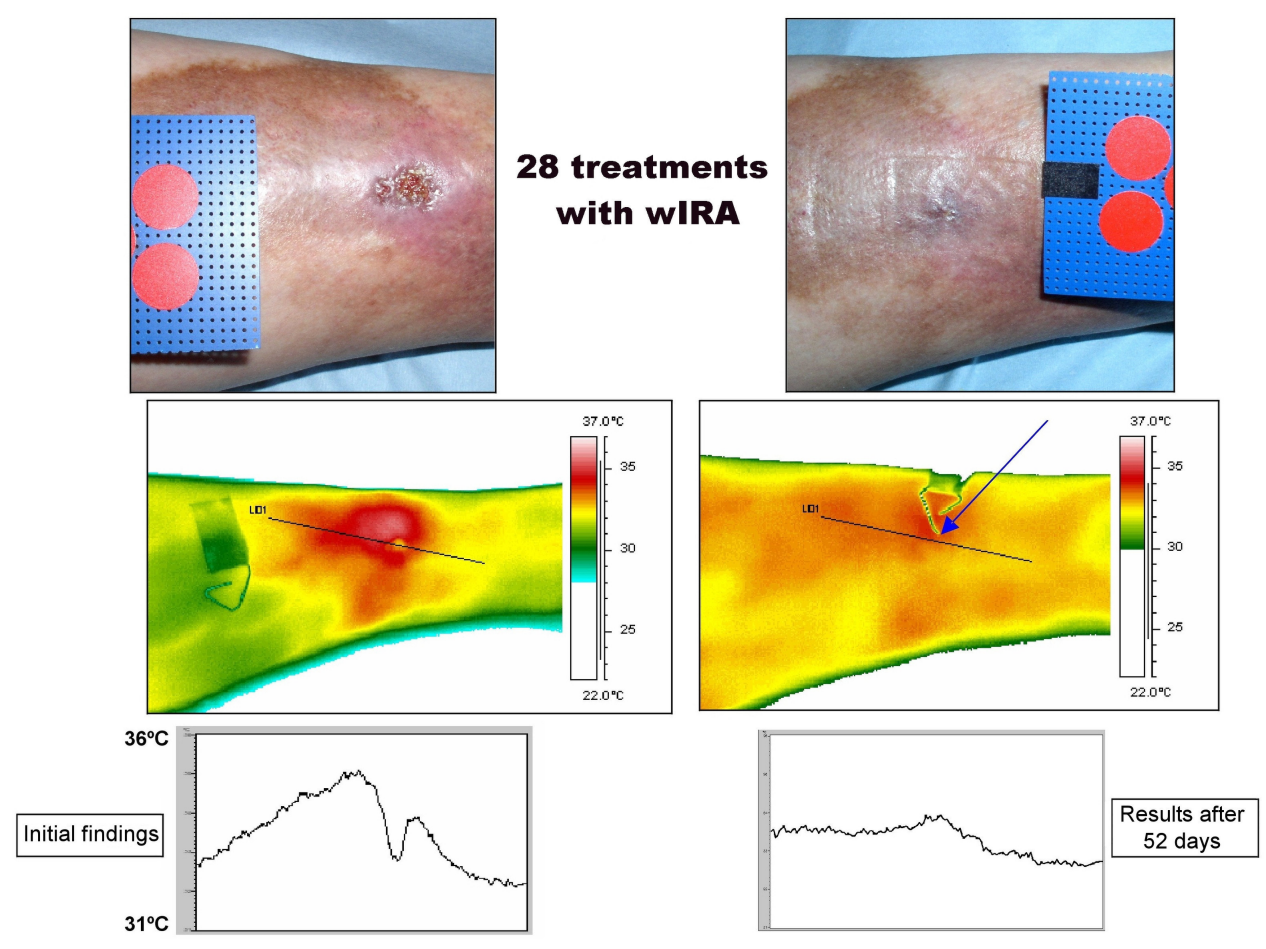
Example of the healing process of a chronic venous stasis ulcer of the lower leg under therapy with wIRA including thermographic images (Study Hillerød/Tromsø) (28 times 30 minutes irradiation with water-filtered infrared-A (wIRA) and visible light (VIS) within 52 days = approximately 7 ½ weeks) with normal view, thermographic image, and temperature profile across the ulcer before therapy (left) and after completion of the course of therapy (right). The arrow in the thermographic image – taken after completion of the course of therapy – points to the place where the wound has been. Diameter of the red circles: 16 mm. (from [4], adapted from [8], [10], [18]).

A further prospective study (Hillerød Hospital, Hillerød, Denmark, in collaboration with the University of Tromsø, Norway) [8], [18] with 10 patients with extensive thermographic investigations showed upon treatment with wIRA(+VIS) (with a maximum of 140 mW/cm² wIRA and 45 mW/cm² VIS) a complete or almost complete wound healing (96–100% reduction of the wound area) of chronic non-healing venous stasis ulcers of the lower legs (initial wound area of 0.42–6.30 cm²) in 7 of the 10 patients and a considerable reduction in the ulcerated area in 2 further patients. There was a pronounced reduction of pain and the required dose of analgesics (Table 1 (Tab. 1)), and a normalisation of the thermographic image (before therapy, typically hyperthermic rim of the ulcer together with a relative hypothermic ulcer base and a temperature difference of up to 4.5°C across the wound [8], [18], see Figure 9 (Fig. 9)). In the 6 patients without accompanying problems, i.e., non-smokers with compression therapy and without peripheral artery occlusive disease, all 6 ulcers healed completely or almost completely. 

Overall, during the course of treatment pronounced improvements of the evaluation of wound healing, the cosmetic result and the effect of irradiation were found. 

Within this study a comparison in one patient (therapy of an ulcer on one leg with wIRA(+VIS), therapy of an ulcer on the other leg with VIS only) showed a clear difference in favour of wIRA [8], [18].

**Figure 10:**
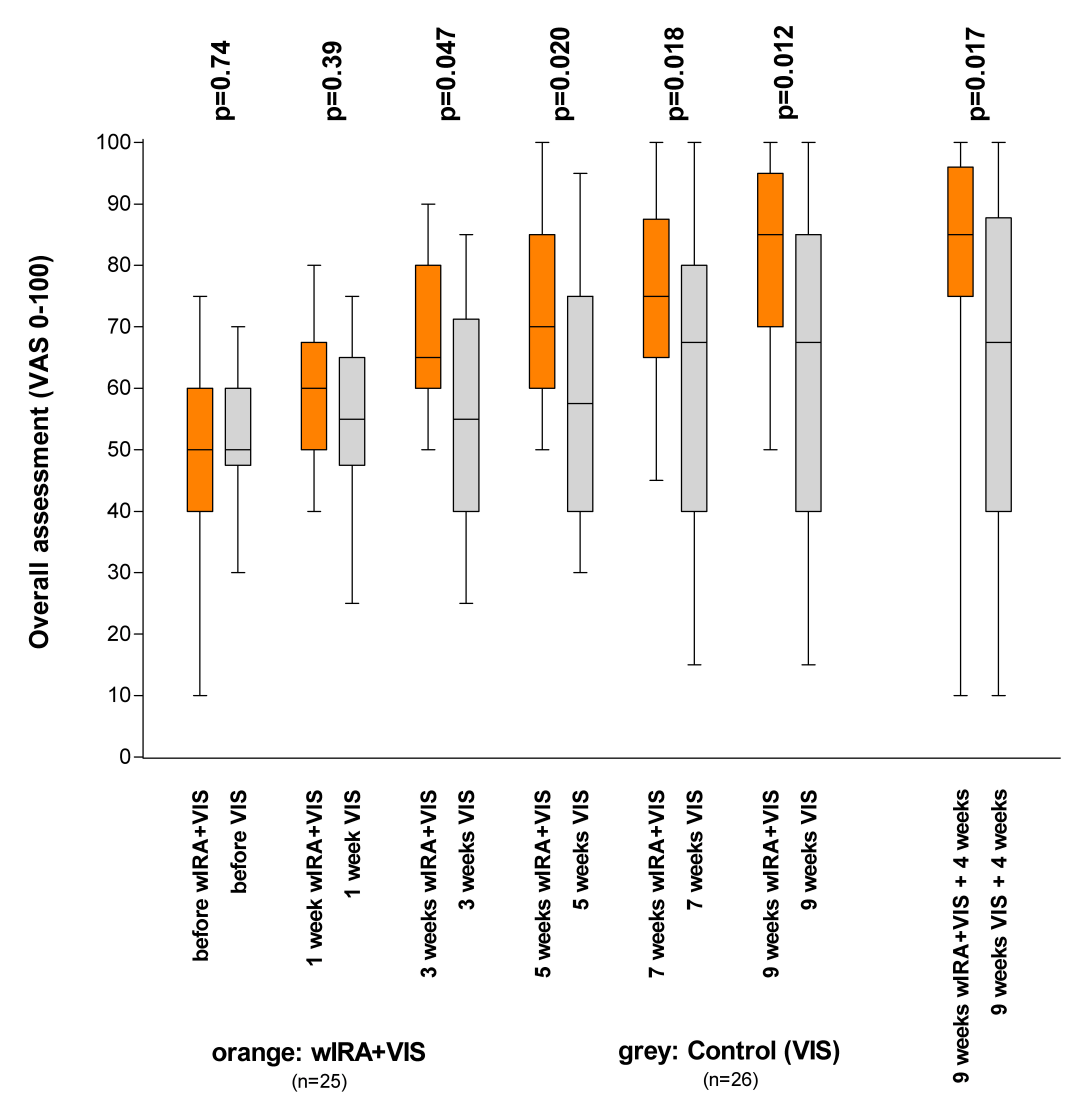
Overall assessment of the wounds in the group with water-filtered infrared-A (wIRA) and visible light (VIS) and in the control group with only visible light (VIS) (chronic venous stasis ulcers of the lower legs, Study Freiburg) (assessed using a visual analogue scale VAS 0–100, 0 = extremely bad, 100 = extremely good; values are presented as minimum, 25%-percentile, median, 75%-percentile and maximum (box and whiskers graph with the box representing the interquartile range; from [65] with kind permission of Wiley, copyright restricted).

In a prospective, randomised, controlled, blinded study (Department of Dermatology, University of Freiburg, Germany), 51 patients with non-healing chronic venous stasis ulcers of the lower legs (initial ulcer area of 1–68 cm²) were treated with compression therapy, wound cleansing and non-adhesive wound dressings and 30 minutes irradiation five times per week over 9 weeks with a further 4 weeks of treatment without irradiation [65]. The group treated with wIRA(+VIS) (maximum of 140 mW/cm² wIRA and 45 mW/cm² VIS) showed, compared to the control group (treated only with VIS), improved overall wound healing (after 9 weeks 85 versus 67.5 on a VAS 0–100, p=0.012, see Figure 10 (Fig. 10)), a greater healing tendency (after 9 weeks in 84% (21 of 25) of the patients compared to 50% (13 of 26), p=0.023), improved granulation (after 9 weeks 90 versus 80 on a VAS 0–100, p=0.036), as well as a tendency towards less exudation, less wound crusts and a more rapid reduction of the wound area (an example of complete wound closure is depicted in reference [65]) (see also Table 1 (Tab. 1)). The limited number of patients who could be recruited within a reasonable time span, the inhomogeneity of wound size with the inclusion of large wounds, the short daily irradiation time (see chapter “Limitations of the studies”) and the limited duration of the irradiation treatment period may all have contributed to missing a significance of the variable “integral of the relative ulcer area for each individual patient over time” in this study. The use of the wIRA radiators by patients at home was found to be easily manageable [65].

### Further wound-related indications for wIRA 

wIRA healed ulcers in patients with morphea [75] and reduced skin hardness and plaques [75], [76], [77]. wIRA can be used for the prophylaxis and therapy of decubitus ulcers [10], [12]. wIRA can be applied in wounds to improve resorption and by this to increase the effects of topically applied substances [12] (level of evidence 1a/1b for intact skin [72], [73]). A combination of wIRA with photodynamic therapy in an anti-infection indication [78], [79], [80] is possible [12], [44].

## Limitations of the studies

In all the studies referred to above the duration of irradiation per day was limited: study Heidelberg: 20 minutes of irradiation twice per day (equals 40 minutes per day); Munich: a single, 20-minute irradiation; Kassel: 30 minutes irradiation daily; Berlin: 30 minutes daily; Basel: 30 minutes three times per week (equals 13 minutes per day); Hillerød/Tromsø: 30 minutes two to five times per week (equals 9–21 minutes per day); Freiburg: 30 minutes five times per week (equals 21 minutes per day). This means all irradiation times lie between 9 and 40 minutes per day, with one exception not higher than 30 minutes per day. From a clinical point of view this is a small amount of daily irradiation time: 

Winkel et al. [5] described in clinical routine use (with some hundred patients having been treated with wIRA) typically two to three times per day 30 minutes, often in total 2 hours per day, seldom up to 5 hours per day. He recommended to irradiate at least 60 minutes per day or markedly longer, e.g. 2–6 hours per day, with moderate irradiance and to continue with wIRA irradiation up to complete wound healing. Longer irradiation times per day result in larger effects. Thus, more frequent and longer lasting irradiations with small irradiances are preferred to shorter lasting irradiations with higher irradiances [5]. It is therefore suggested that the studies described above underestimate the positive effects of wIRA. For this reason the recommended daily irradiation times presented in the section “Application of wIRA” are much higher than in the described studies. 

## Conclusions and perspectives

Positive effects of wIRA on wound healing were described in 7 prospective studies (of these six randomised controlled trials (RCTs), evidence level 1a/1b) and in the everyday clinical routine (see Table 1 (Tab. 1)).

- wIRA is a useful therapeutic option recommended for the treatment of acute and chronic wounds [4], [7], [8], [10], [12], [18], [37], [65], [70], [74]. wIRA can considerably alleviate pain with a substantially reduced need for analgesics (52–69% less) [12], [18], [37]. Wound exudation and inflammation are also diminished [12], [18], [37]. Wound healing, clinical impression, and cosmetic result are markedly improved [4], [12], [18], [37].
- Considering the results presented, it seems clinically reasonable to apply wIRA before and after operations. wIRA is a positive adjunct to pre- or postoperative routine administration of antibiotics (under certain conditions wIRA might even replace antibiotics, although this has not yet been tested) [4], [12], [37], [70].
- In chronic ulcers of the lower legs wIRA is a positive adjunct to causal therapies for wounds of various aetiologies [4], [8], [10], [12], [18], [65].
- wIRA can be used to improve the resorption of topically applied substances [12], [72], [73].

Some perspectives for the implementation of wIRA in wound-related indications have only been described casuistically or in smaller series:

- wIRA preoperatively (e.g., over 1–2 weeks) for preconditioning of the sites of removal and transplantation of skin grafts, transplants and split-skin grafts.
- wIRA postoperatively to promote wound healing and to reduce pain, exudation, inflammation and infection at the mentioned locations [4], [12].
- wIRA as a non-invasive alternative to the punction of wound seromas and wound haematomas and to wound revisions [4], [7], [12].
- wIRA for prophylaxis and therapy of decubitus ulcers [10], [12].
- wIRA with small irradiances and starting with short irradiation times to treat the Complex Regional Pain Syndrome CRPS [12].

## Notes

### Acknowledgements

We are grateful to Dr rer. med. Dipl.-Math. Hanns Ackermann, the deputy head of the Institute of Biostatistics and Mathematical Modelling of the Johann Wolfgang Goethe University Clinic Frankfurt/Main, for statistical advice, and Assistant Prof. Dr. Debra Bickes-Kelleher of the Johannes Gutenberg University Mainz for assistance in translating the manuscript. 

### Contributors

GH provided the concept of the paper, MH and JBM contributed the expertise of their own clinical trials. All authors contributed to each draft of the paper.

### Conflicts of interest

GH is working for the Dr. med. h.c. Erwin Braun Foundation, Basel, Switzerland, a charitable, non-profit Swiss scientific foundation approved by the Swiss Federal Administration. MH received a research grant from this foundation. The foundation was not involved in any content- or decision-related aspect of the review. None of the authors is or was employed by a commercial company or received fees or grants by a commercial company. Therefore, the authors declare that no conflicts of interest exist according to the guidelines of the International Committee of Medical Journal Editors. 

## Figures and Tables

**Table 1:**
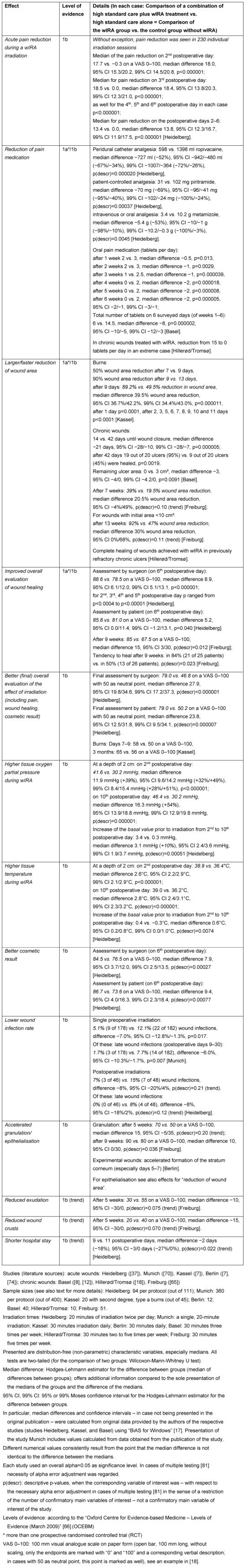
Levels of evidence for the effects of water-filtered infrared-A (wIRA) in acute and chronic wounds in humans
